# Danlu tongdu tablets: Preclinical safety evaluation and mechanism of hepatotoxicity

**DOI:** 10.3389/fphar.2022.1023379

**Published:** 2022-11-03

**Authors:** Haijing Zhang, Yifei Yang, Feifei Guo, Rong He, Shuangrong Gao, Chunyu Cao, Chunhui Zhao, Bing Xia, Qihua Xu, Ping Gong, Lifang Wang, Ping Su, Ting Liu

**Affiliations:** Institute of Chinese Material Medica, China Academy of Chinese Medical Sciences, Beijing, China

**Keywords:** Danlu tongdu tablets, acute toxicity, chronic toxicity, hepatotoxicity, transcriptome analysis, cytochrome P450 enzyme

## Abstract

Danlu tongdu tablets (DLTD) is a listed Chinese patent medicine collected in the Pharmacopoeia of the People’s Republic of China (version 2020). This prescription has been applied in clinics in China for lumbar spinal stenosis and lumbosacral disc herniations. The wide application of Danlu tongdu in therapy has raised some clinical adverse reactions, such as significant elevation of alanine transaminase (ALT) and aspartate transaminase (AST) in individual patients after use. The present study aimed to investigate the safety of Danlu tongdu and analyze its adverse effects on the liver. The maximum feasible dose (MFD) was used to carry out the acute toxicity tests. Mortality, adverse effects, body weight and food consumption were recorded for up to 14 days post treatment. In the 6-month chronic toxicity test, sprague-dawley rats were randomly divided into four groups according body weight, the experimental groups were administrated to rats at the concentrations of 1.67, 3.34 and 6.67 g/kg/day, whereas the control group was received the ultrapure water (vehicle) only, 10 ml/kg, once a day. The animal’s body weight, food consumption was monitored weekly. In addition, their hematological and biochemical parameters, body and organ weights and histopathology, were all measured at specific observation time points. Additionally, we further explored the adverse effects mechanism of Danlu tongdu on the liver through transcriptome analysis. No deaths or substance-relative toxicity were observed in the acute toxicity study or the 6-month chronic toxicity study with doses of 1.67 g/kg and 3.34 g/kg, respectively. We found that mild hypertrophy and hyperplasia of hepatic interlobular bile ducts were detected in some rats with doses of 6.67 g/kg after repeated oral administration of Danlu tongdu for 13 and 26 weeks, but the above changes in liver were reversible. The results of transcriptome sequencing showed that Danlu tongdu had a significant effect on cytochrome P450 enzymes in rat liver, especially cytochrome P450 1 (CYP1) subtype. Therefore, the toxic target organ of Danlu tongdu is the liver and the mechanism of mild liver injury is closely related to the up-regulation of cytochrome P450 1A1 (CYP1A1) and cytochrome P450 1A2 (CYP1A2) expression.

## 1 Introduction

Danlu tongdu tablets (DLTD), a listed Chinese patent medicine consisting of five traditional Chinese drugs, include Salviae miltiorrhizae, Astragali radix, Eucommiae cortex, Corydalis rhizoma and Cervi cornus colla (Li et al., 2013; Li and Zhao, 2013; Commission, 2020). This prescription has the functions of promoting blood circulation, removing obstruction in channels, tonifying kidney and supplementing qi, which is used in clinical treatment of lumbar spinal stenosis with pattern of qi deficiency and blood stasis, lumbosacral disc herniations, intermittent claudication, pain in waist and leg, limitation of movement, acid distension in lower limbs, *etc.* The clinical effect is remarkable(Pan, 2010; Guo, 2011a; Guo, 2011b; Huang, 2012; Liu et al., 2012; Zhou, 2012; Chen, 2015; Duo, 2015; Yan et al., 2019; Wei, 2022).

Cervi cornus colla in DLTD has the function of enriching blood and nourishing essence. Pharmacological experiments have proved that it has many functions, such as anti-inflammation and analgesia, tonifying blood and promoting blood circulation, anti-osteoporosis, promoting cartilage and osteoblast proliferation (Lu et al., 2021). Salviae miltiorrhizae in DLTD has the function of promoting blood circulation and resolving stasis and the chemical components were mainly classified as lipid-soluble compounds tanshinones and water-soluble compounds salvianolic acids. The pharmacological studies, including anti-platelet aggregation, anti-thrombosis, improve hemorheology, improve microcirculation, anti-inflammatory, lipid metabolism, have been widely performed (Fan et al., 2010; Wu et al., 2018). The main pharmacodynamic material base of Corydalis rhizoma is the total alkaloid. The pharmacological activity studies showed that Corydalis rhizoma has good sedative, analgesic, antiarrhythmic and antihypertensive effects (Feng et al., 2018). The chemical components of Eucommiae cortex mainly included lignans, iridoids, phenylpropanoids, flavonoids and other active ingredients. Modern pharmacological actions included regulating bone metabolism, tonifying kidney, enhancing immunity function, and so on (Wang et al., 2017). The Astragali radix is mainly composed of polysaccharides, triterpenes and flavonoids. It has pharmacological effects such as enhancing and regulating body immunity, anti-aging and anti-fatigue (Zhang et al., 2012). The whole prescription changes the microcirculation of the lesion site, protects the compressed spinal cord from damage, and promotes the functional recovery of the injured nerve.

With the widespread use of DLTD, 4 cases of drug-induced liver injury caused by DLTD have been reported, including 1 case in 2011 (Chen, 2011), 1 case in 2017 (Zhao and Wang, 2017) and 2 cases in 2018 (Zhang and Leng, 2018). All patients had nausea, general fatigue, pathological increase of liver function indexes such as alanine aminotransferase (ALT), aspartate transaminase (AST), alkaline phosphatase (ALP), total bilirubin (TBIL) and direct bilirubin (DBIL), and all indexes of liver function recovered after drug withdrawal. In the drug description, the adverse reaction only indicates that “rash occurs in individual patients”, and does not indicate that long-term application may have potential liver function damage. In addition, there are no reports on the preclinical safety evaluation and hepatotoxicity of DLTD. In order to further determine the safety of DLTD in clinical use, especially whether it has adverse effects on the liver, we evaluated DLTD from three aspects: liver damage marker detection, histopathological examination and transcriptomic analysis, hoping to obtain more information and provide certain data for the safety of clinical use.

## 2 Materials and methods

### 2.1 Test substances

Danlu tongdu tablets are the listed Chinese patent medicine collected in the Pharmacopoeia of the People’s Republic of China (version 2020, volume 1, page 717–718). In order to facilitate the preparation of the drug liquid, the test substances were the powder of Danlu tongdu tablets without magnesium stearate and other auxiliary materials. The powder of Danlu tongdu tablets (batch No. 200303–04) was supplied by Henan Lingrui Pharmaceutical Co., Ltd. (Xinyang, Henan, China) and prepared as follows: 1,500 kg of Astragali radix [Leguminosae; Astragalus membranaceus (Fisch.) Bge.var.mongholicus (Bge.) Hsiao; Astragalus membranaceus (Fisch.) Bge.] and 1,500 kg of Eucommiae cortex (Eucommiaceae; Eucommia ulmoides Oliv.) were boiled with water for 3 times, 1 h each time, combined the water decoction, filtered, filtered liquid concentrated to the right amount; 501 kg of Cervi cornus colla (a gelatin made of deer antlers by boiling and concentrating process) melted by heat; 1,500 kg of Salviae miltiorrhizae (Labiatae; Salvia miltiorrhiza Bge.) and 999 kg of Corydalis rhizome (Papaveraceae; Corydalis yanhusuo W.T.Wang) were refluxed with 70% ethanol for 3 times, 1 h each time, the extracting solution was merged, filtered, and the filtrate was concentrated to an appropriate amount under reduced pressure; finally, the above-mentioned ointment was combined, dried and crushed. The relative share of Astragali radix, Eucommiae cortex, Salviae miltiorrhizae, Corydalis rhizome, Cervi cornus colla are 3:3:3:2:1. The drug extract ratio was 24.2% and per Gram of powder was equivalent to 4.11 g of crude drug. The moisture content of the powder was 3.3%. Thin-layer chromatography was used for differentiating the Salviae miltiorrhizae, Astragali radix and Corydalis rhizome in the prescription. The content of astragaloside per Gram of powder was 0.91 mg.

### 2.2 Experimental animals

40 specific pathogen-free (SPF) ICR mice (half of each sex) were obtained from Beijing Vital River Laboratory Animal Technology Co., Ltd. (Beijing, China) with the confirmation number SCXK (Jing) 2016–0006. Mice were housed in polypropylene cages at an ambient temperature of 23°C (±3°C) and 40–70% relative humidity, with a 12:12 h light/dark cycle. Animals were provided with commercial food pellets and water *ad libitum* unless stated otherwise. The animals were allowed 3 days of acclimatization before the commencement of experimental procedures.

200 SPF rats (100 male and 100 female) were obtained from Beijing Vital River Laboratory Animal Technology Co., Ltd. (Beijing, China) with the confirmation number SCXK (Jing) 2016–0006. Rats were housed in polypropylene cages at an ambient temperature of 23°C (±3°C) and 40–70% relative humidity, with a 12:12 h light/dark cycle. Animals were provided with commercial food pellets and water *ad libitum* unless stated otherwise. The animals were allowed 7 days of acclimatization before the commencement of experimental procedures.

The animal experiments were conducted at Traditional Chinese Medicine Safety Evaluation Center, Institute of Chinese Materia Medica China Academy of Chinese medical science (Beijing, China), according to good laboratory practice (GLP) guidelines, and were authorized by Committee of Welfare and Ethics for Experimental Animals. The approval numbers for acute toxicity study and chronic toxicity studies were 2020A023 of and 2020A028, respectively.

### 2.3 Safety evaluation of Danlu tongdu tablets

#### 2.3.1 Acute toxicity study

For the acute toxicity study, we followed the technical guidelines for single dose toxicity study of pharmaceuticals published by the National Medical Products Administration (NMPA, 13 May 2014). The maximum feasible dose (MFD) was used to carry out the acute toxicity tests. The concentration of DLTD solution was 0.89 g/ml, which is the maximum concentration that could be given to mice by gavage. The dose volume was 40 ml/kg, once within 24 h. Therefore, the dose of DLTD given to each mice was 35.60 g/kg, and was equivalent to 427 times of the proposed clinical dose. 40 mice were randomly divided into two groups of 20 (equally divided by sex): control group (treated with ultrapure water) and DLTD treated group (35.60 g/kg). All mice were fasted overnight prior to the intragastric administration. The mice were closely monitored for 2 h after the dose administration, both in the morning and afternoon. The day when the mice were exposed to the test substance was recorded as D0. General health observations (daily), symptoms of poisoning (daily) and mortality (daily) were monitored and registered for 2 weeks. Body weights and food consumption were measured after administration on days 1, 2, 3, 5, 7, 10 and 14. The observation period of 2 week’s post treatment was recorded as D1 to D14. The mice were sacrificed by cervical dislocation and examined for histopathological changes at D14.

#### 2.3.2 6-Month chronic toxicity study

The 6-month chronic toxicity of DLTD was evaluated according to the technical guidelines for repeated dose toxicity study of pharmaceuticals (NMPA, 13 May 2014). 200 rats were randomly divided into four groups of 50 (half of each sex): control group (treated with ultrapure water), low-dose group (treated with 1.67 g/kg DLTD), medium-dose group (treated with 3.34 g/kg DLTD) and high-dose group (treated with 6.67 g/kg DLTD). Rats were given the above dose of liquid medicine by gavage every morning for 6 months and the corresponding period was recorded as D1-D183. In addition, there was a 4-week observation period after the final administration which was recorded as rD1 to rD29. General health observations were conducted daily, and the weights and food consumption for each survival rat were recorded twice per week for the first 2 weeks, and then once per week. The volume of DLTD was adjusted according to the weights of the rats.

In the early stages of administration period (D28), 10 rats (half of each sex) among each group were fasted for overnight, and their urine samples were collected. On the next day (D29), 40 rats were subjected general anesthesia by intramuscular injection of zoletil^®^50 at a dose of 40 mg/kg in male rats or 30 mg/kg in female rats. After anesthesia, blood was collected from the abdominal aorta until the animals died. And the collected blood samples were applied to hematologic analysis (EDTA-K2 tubes), biochemistry analysis (dry tubes) and coagulation tests (sodium citrate tubes). Vital organs, including the heart, liver, kidney and so on, were excised, weighed and fixed in 10% formaldehyde solution for histopathological examination after euthanasia. The femur marrow was used for the bone marrow smear. Similarly, rats were sacrificed and examined at the middle of the administration period (D92, 20 male and 20 female), at the end of the administration period (D182, 40 male; D183, 40 female) and the end of the recovery period (rD29, 20 male and 20 female), respectively.

##### 2.3.2.1 Hematologic analysis

For the hematologic analysis, the samples were collected in EDTA-K2 tubes and analyzed on the day of preparation. The red blood cell count (RBC), hemoglobin (HGB), hematocrit level (HCT), mean corpuscular volume (MCV), mean corpuscular hemoglobin (MCH), mean corpuscular hemoglobin concentration (MCHC), white blood cell count (WBC), neutrophil ratio (NEUT%), lymphocyte ratio (LYM%), monocyte ratio (MONO%), eosinophil ratio (EOS%), basophil ratio (BASO%), platelet count (PLT), and reticulocyte ratio (RET%) were examined using an sysmex XN-1000 automated hematology analyzer (Sysmex, Japan). Samples collected with sodium citrate-containing tubes were used for the coagulation test. The prothrombin time (PT) and activated partial thromboplastin time (APTT) were measured using a ACL TOP 500 automatic coagulation analyzer (Werfen, Spain).

##### 2.3.2.2 Blood biochemistry analysis

For the blood biochemistry analysis, the blood samples collected with dry tubes were rested for 30 min at room temperature. Then the samples were centrifuged at 3,000 rpm for 15 min and the supernatant was carefully transferred to another cleaning tube. Creatine kinase (CK), creatine kinase MB isoenzyme (CK-MB), lactic de-hydrogenase (LDH), alanine transaminase (ALT), aspartate transaminase (AST), alkaline phosphatase (ALP), γ-glutamyltransferase (GGT), total bilirubin (TBIL), total protein (TP), albumin (ALB), creatinine (CRE), urea nitrogen (UREA), glucose (GLU), total cholesterol (CHOL) and triglycerides (TG) were detected using a toshiba TBA-120FR analyzer (Toshiba, Japan), while potassium (K^+^), sodium (Na^+^), chloride (Cl^−^) were detected using a medica Easylyte analyzer (Medica, United States).

##### 2.3.3.3 Histological analysis

The fixed organs were dehydrated in gradient ethanol, vitrified, and embedded in paraffin. Sections (3 μm thick) were cut with an RM2135 rotary microtome sinica (Leica, Germany) and stained with hematoxylin and eosin. Histological observations were performed using olympus BX51 microscope (Olympus, Japan) at 200 magnification to evaluate the degree of pathological alteration. Pathological evaluation of the tissue sections was performed by an experienced pathologist.

### 2.4 RNA sequencing assay

At the end of administration, four male rats were selected from the control group and the high-dose group respectively for transcriptome analysis. The selection criteria were random selection from the control group, the four rats selected from the high-dose group showed pathological changes in the liver. For the whole transcriptome analysis, total RNA were extracted from the liver isolated from the control group or high-dose group rats at the end of the administration period. Each group of the RNA-sequencing assay used four samples. The RNA-sequencing assay included the cDNA library construction, library purification, transcriptome sequencing and GO and KEGG enrichment analyses. All experiments were finished according to the protocols of the Tianjin Novogene Bioinformatic Technology Company’s Instructions. GO and KEGG enrichment analyses revealed the functional roles of differentially expressed genes (DEGs). The threshold was 0.05 to test the hypergeometric distribution of the default enrichment results.

### 2.5 Quantitative real-time PCR

At the end of the administration, 10 male rats in the control group and 10 male rats in the high dose group were used for quantitative real-time PCR and western blot detection below, and the sample numbers were set as control 1–10 and DLTD 11–20. The total RNA of the liver was extracted with trizol reagent. The revert aid first strand cDNA synthesis kit (Thermo) was used to reverse-transcribe RNA to cDNA. quantitative real-time PCR was performed with the GoTaq^®^ qPCR master mix (Promega). The beta-actin value was used as the normalized expression control. The sequences of primers are shown in Table 1.

**TABLE 1:** Primers to amplify the target sequences.

Target gene	Forward primer (5’→3′)	Reverse primer (5’→3′)
CYP1A1	TTT​AAT​GTG​TCA​CCA​GGC​GAG​AAG​G	TGG​AGA​TGC​TGA​GGA​CCA​GAA​GAC
CYP1A2	GGT​GGT​GGA​ATC​GGT​GGC​TAA​TG	CTT​GCT​GCT​CTT​CAC​GAG​GTT​GAG
CYP2B1	TTT​GGT​GGA​GGA​ACT​GCG​GAA​ATC	AGG​AAC​TGG​CGG​TCT​GTG​TAG​TC
CYP2C11	AGC​ACA​ATC​CGC​AGT​CTG​AGT​TTA​C	AGC​AGC​AGC​AGG​AGT​CCA​TAC​C
CYP2D1	TCC​TCT​TCT​TCA​CCT​GCC​TCC​TG	AGT​CCT​TGC​TCC​CGT​ACC​ACA​G
CYP2E1	CAA​TCT​TGT​CCC​TTC​CAA​CCT​ACC​C	CTG​GAT​CTG​GAA​ACT​CAT​GGC​TGT​C
CYP3A1	CGT​TCA​CCA​GTG​GAA​GAC​TCA​AGG	ACT​TCT​TTC​ACA​GGG​ACA​GGT​TTG​C
β-actin	CCA​GAT​CAT​GTT​TGA​GAC​CTT​CAA	GTG​GTA​CGA​CCA​GAG​GCA​TAC​A

CYP1A1 cytochrome P-450 1A1, 1A2, 1B1, 2C11, 2D1, 2E1, 3A1.

### 2.6 Western blot

Proteins were obtained from tissue lysates with the RIPA buffer. The bicinchoninic acid (BCA) assay kit was used to determine the concentration of the protein. The total lysates were loaded and separated *via* Tris-SDS PAGE and then transferred to polyvinylidene difluoride (PVDF) membranes. After blocking with 5% fat-free milk powder, specific primary antibodies were incubated with PVDF membranes overnight. Then, HRP-conjugated secondary antibody was added, and the expression of the protein was examined with the ECL plus reagents.

### 2.7 Statistical analysis

The experimental data were analyzed using SPSS 20.0 statistical software, and the data were expressed as mean ± standard deviation (SD). The weekly body weight and food consumption were analyzed by repeated measure one-way analysis of variance (ANOVA). The hematological indicators, serum biochemical indicators, visceral body ratio coefficient and other indicators were assessed by ANOVA, according to the results of homogeneity test of variance, Dunnett method is used for homogeneous variance, and Dunnett’s T3 method is used for uneven variance. *p*-values less than 0.05 were considered to be significant.

## 3 Results

### 3.1 Acute toxicity study of Danlu tongdu tablets

**FIGURE 1:**
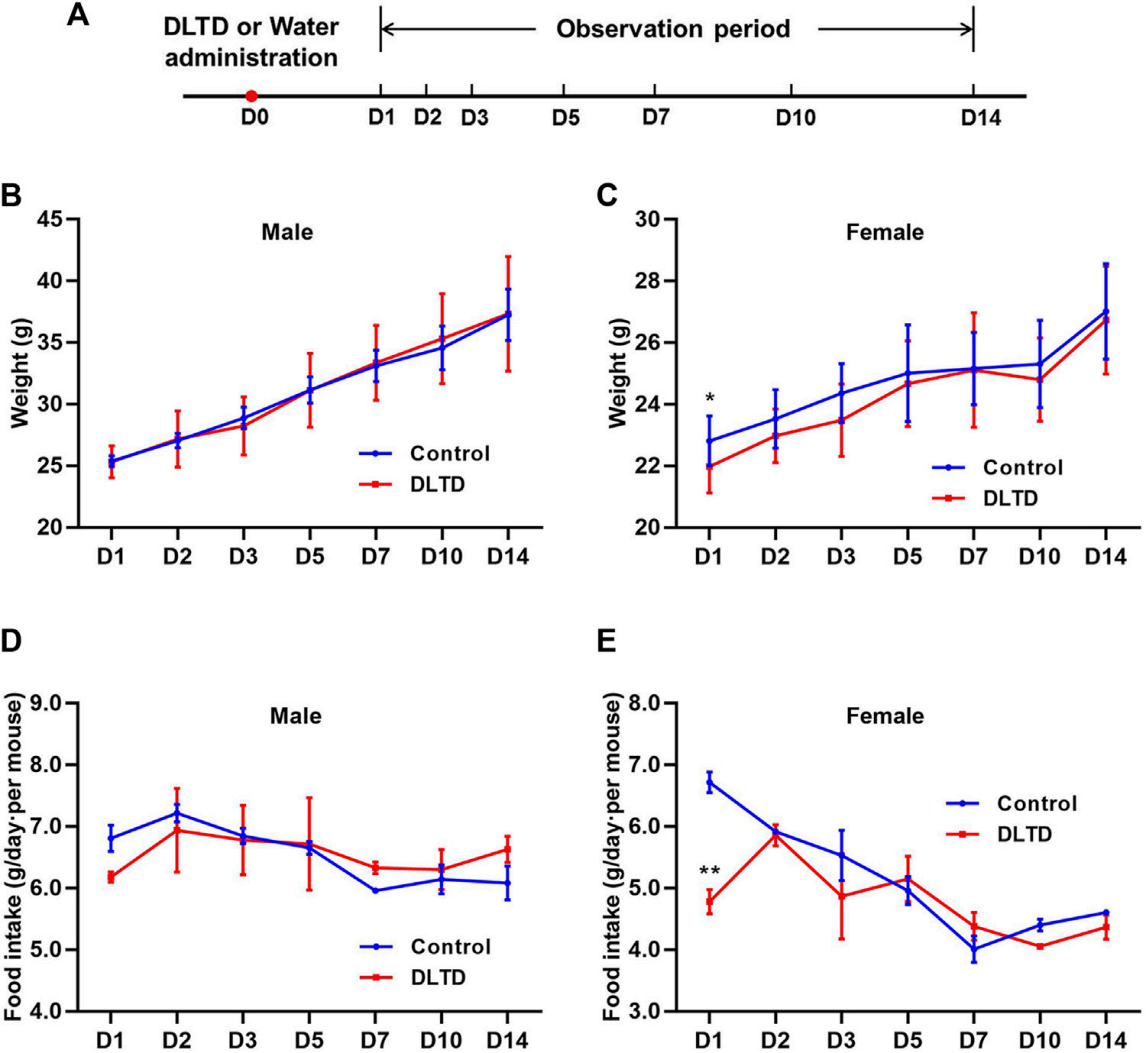
Body weight and food consumption of mice in acute toxicity studies. **(A)** Timeline of the acute toxicity study. Weights of male mice **(B)** and female mice **(C)** in the acute toxicity study. Food consumption of male mice **(D)** and female mice **(E)** in the acute toxicity study. Statistical analysis was performed using the *t*-test. *p < 0.05, **p < 0.01 *versus* the control.

After 14-day observation period, no deaths occurred in mice and the major adverse reaction was transient diarrhea with the DLTD treatment, the incidence of diarrhea was 70% (♂9/10♀5/10). Body weight and food consumption was measured at D1, D2, D3, D5, D7, D10 and D14 after administration. Only on the first day after administration, the body weight and food consumption of female animals decreased significantly, and there was no statistically significant difference between the DLTD-treated and non-treated groups at other time points (Figures 1A–E). This transient change may be related to the thicker and larger volume of liquid medicine. At the end of the acute administration evaluation, pathological examination showed no obvious lesions or abnormalities in the volume, color, or texture of isolated organs due to the treatment with DLTD (data not shown).

### 3.2 Chronic toxicity study of Danlu tongdu tablets

**FIGURE 2:**
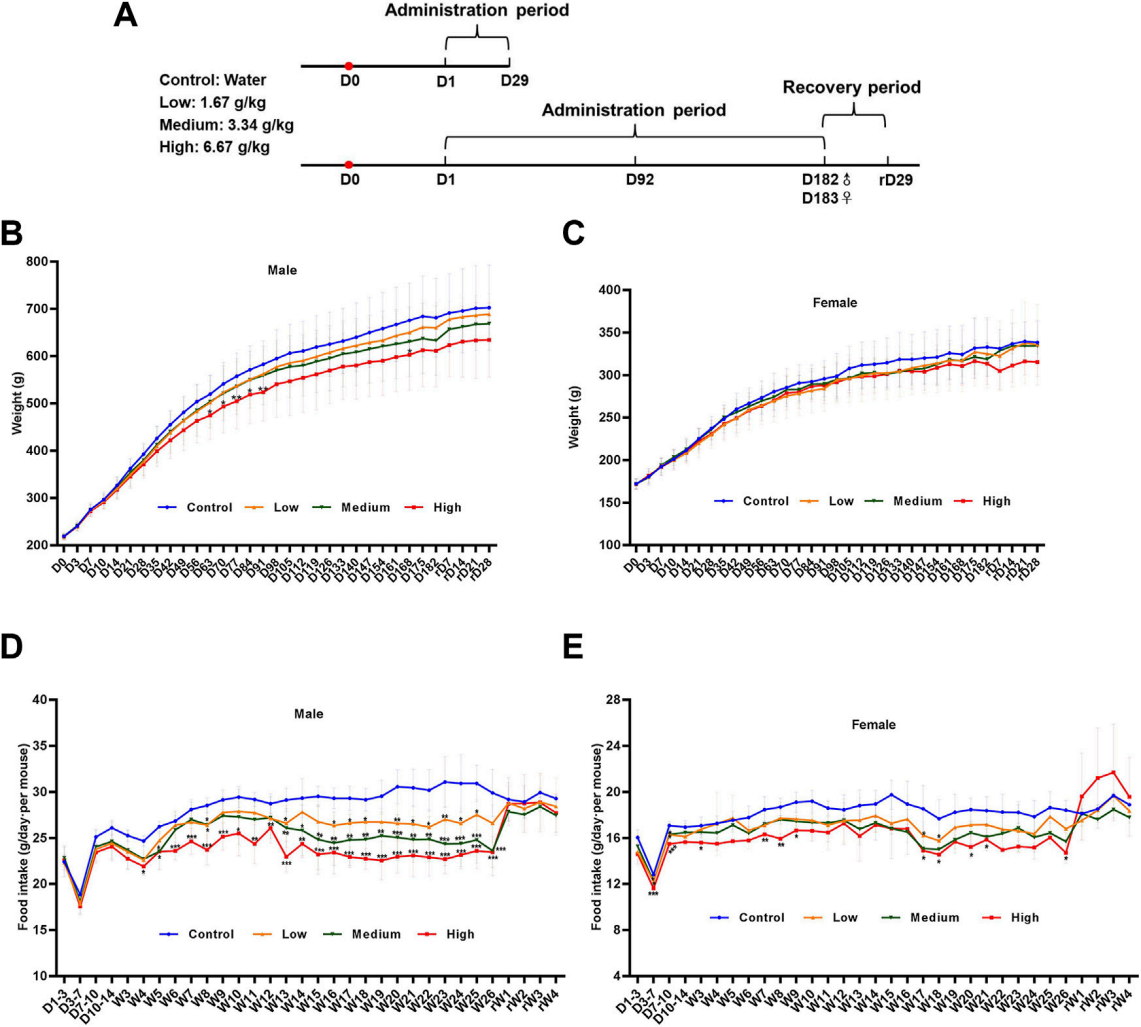
Body weight and food consumption of rats in chronic toxicity studies. **(A)** Timeline of the 6-month chronic toxicity study. Weights of male rats **(B)** and female rats **(C)** in the 6-month chronic toxicity study. Food consumption of male rats **(D)** and female rats **(E)** in the 6-month chronic toxicity study. Statistical analysis was performed using the one-way ANOVA. *p < 0.05, **p < 0.01, ***p < 0.001 *versus* the control.

We performed ophthalmology and urine examinations on rats at the mid-term administration period (D91), the end of the administration period (D181 and D182) and the recovery period (rD28), and the hematology, blood biochemical assay and histopathological examinations were performed at D29, D92, D182, D183, and rD29 (Figure 2A). From the chronic toxicity testing, there was no abnormal clinical signs or deaths occurred in rats with the DLTD treatment.

#### 3.2.1 Body weight and food consumption

As expected, the rats gained weight with time during the administration and recovery period. In the male rats, body weights decreased significantly in the high-dose group on days 63, 70, 77, 84, 91 and 168 compared with that in the control group. At the same time, body weights showed no significant differences in the low- and mid-dose group. In the female rats, there was no significant difference between the low, medium, high-dose group and the control group. Body weight curves for male and female rats are shown in Figures 2B,C. Although statistically significant intergroup differences in mean food consumption were seen in different time, which could be considered as a normal change because rats were administered 15 ml/kg liquid medicine twice within a day (Figures 2D,E).

#### 3.2.2 Hematology

Hematological changes were evaluated five times: on Day 29, Day 92, Day 182 and Day 183 of the study and D29 after recovery. The effect of oral administration DLTD on hematological parameters compared with control group is presented in Table 2. In hematological parameters, a significant increase in platelet count was observed in low-dose (*p* < 0.05), in mid-dose (*p* < 0.05) and in high-dose males (*p* < 0.01) on Day 29. At the same time, reticulocyte ratio were significantly increased in high-dose group (*p* < 0.01). On Day 92 significant increase were observed in hematocrit level in low-dose (*p* < 0.05), in mid-dose (*p* < 0.05) and in high-dose males (*p* < 0.01), meanwhile, in hemoglobin in low-dose (*p* < 0.01) and in mid-dose males (*p* < 0.05). Red blood cell count was also elevated in mid-dose (*p* < 0.05) and high-dose males (*p* < 0.01) on Day 182. In high-dose females, hemoglobin were significantly increased after 4-week recovery (*p* < 0.05). There was no abnormality in hematological parameters of male rats in each dose group in the recovery period.

**TABLE 2:** Hematological parameters in rats treated with DLTD.

Parameter	Male				Female			
	Control	Low	Medium	High	Control	Low	Medium	High
D29								
RBC(10^12^/L)	8.30 ± 0.28	8.22 ± 0.26	8.41 ± 0.22	8.20 ± 0.39	7.87 ± 0.24	7.98 ± 0.56	7.91 ± 0.28	7.82 ± 0.29
HCT(%)	47.3 ± 1.4	46.8 ± 1.3	48.1 ± 1.1	47.2 ± 2.7	44.7 ± 1.1	44.5 ± 2.0	45.5 ± 1.4	44.8 ± 1.5
MCV(fL)	57.0 ± 1.1	56.9 ± 1.0	57.3 ± 0.7	57.5 ± 2.0	56.7 ± 1.6	55.8 ± 1.6	57.6 ± 2.4	57.4 ± 1.0
HGB(g/dL)	15.4 ± 0.6	15.4 ± 0.6	15.8 ± 0.3	15.4 ± 0.8	15.0 ± 0.5	15.4 ± 0.4	15.4 ± 0.4	15.1 ± 0.4
MCH(pg)	18.6 ± 0.4	18.6 ± 0.4	18.8 ± 0.2	18.8 ± 0.6	19.1 ± 0.5	19.4 ± 1.2	19.4 ± 0.6	19.4 ± 0.2
MCHC(g/dL)	32.62 ± 0.31	32.78 ± 0.50	32.92 ± 0.40	32.72 ± 0.34	33.70 ± 0.31	34.70 ± 1.25	33.76 ± 0.36	33.74 ± 0.40
PLT(10^9^/L)	955 ± 66	1,068 ± 91*	1,045 ± 62*	1,120 ± 35**	1,061 ± 55	1,065 ± 208	1,075 ± 76	1,121 ± 65
WBC(10^9^/L)	4.19 ± 0.81	4.90 ± 1.94	4.06 ± 0.77	4.45 ± 0.86	4.55 ± 0.52	4.37 ± 1.77	4.83 ± 0.85	5.06 ± 0.87
LYM%(%)	80.7 ± 9.8	82.3 ± 2.9	76.8 ± 6.8	74.9 ± 5.2	80.7 ± 5.5	85.7 ± 3.9	84.4 ± 1.1	79.8 ± 3.0
NEUT%(%)	15.5 ± 8.1	13.9 ± 2.0	18.8 ± 5.7	19.7 ± 3.9	14.4 ± 4.3	9.3 ± 3.5*	11.0 ± 1.0	15.2 ± 3.9
EOS%(%)	2.7 ± 1.7	2.6 ± 1.5	3.0 ± 1.6	3.6 ± 1.4	3.5 ± 1.0	3.9 ± 1.2	3.3 ± 0.2	3.2 ± 0.6
BASO%(%)	0.0 ± 0.0	0.0 ± 0.0	0.0 ± 0.0	0.0 ± 0.0	0.0 ± 0.0	0.0 ± 0.0	0.0 ± 0.0	0.0 ± 0.0
MONO%(%)	0.9 ± 0.2	1.0 ± 0.2	1.3 ± 0.4	1.6 ± 0.7	1.2 ± 0.6	0.9 ± 0.3	1.1 ± 0.3	1.7 ± 0.9
RET(%)	2.51 ± 0.27	2.72 ± 0.59	2.75 ± 0.39	3.61 ± 0.55**	2.93 ± 0.76	2.16 ± 0.68	2.72 ± 0.72	2.77 ± 0.43
D92								
RBC(10^12^/L)	8.21 ± 0.28	8.54 ± 0.72	8.67 ± 0.35	9.06 ± 0.28	8.10 ± 0.27	7.88 ± 0.57	8.26 ± 0.40	7.94 ± 0.40
HCT(%)	44.4 ± 0.6	47.2 ± 1.6*	47.3 ± 1.8*	47.9 ± 1.7**	45.3 ± 1.4	43.7 ± 2.5	46.0 ± 1.7	44.9 ± 1.7
MCV(fL)	54.1 ± 1.2	55.4 ± 3.2	54.5 ± 0.9	52.8 ± 1.8	55.9 ± 1.1	55.5 ± 1.1	55.7 ± 1.1	56.6 ± 1.0
HGB(g/dL)	14.2 ± 0.2	15.1 ± 0.2**	15.2 ± 0.5*	15.2 ± 0.7	14.7 ± 0.3	14.2 ± 1.0	14.9 ± 0.4	14.5 ± 0.7
MCH(pg)	17.3 ± 0.5	17.8 ± 1.4	17.5 ± 0.2	16.8 ± 0.7	18.1 ± 0.5	18.1 ± 0.3	18.1 ± 0.4	18.3 ± 0.3
MCHC(g/dL)	31.94 ± 0.30	32.04 ± 0.62	32.16 ± 0.37	31.80 ± 0.29	32.38 ± 0.88	32.56 ± 0.51	32.48 ± 0.23	32.24 ± 0.34
PLT(10^9^/L)	1054 ± 145	827 ± 412	919 ± 69	973 ± 77	877 ± 71	991 ± 103	998 ± 149	1022 ± 103
WBC(10^9^/L)	4.22 ± 0.98	4.03 ± 0.91	4.15 ± 0.29	3.63 ± 0.34	2.85 ± 0.71	4.28 ± 2.36	3.26 ± 0.76	3.51 ± 1.00
LYM%(%)	70.2 ± 3.1	69.5 ± 10.2	61.4 ± 14.7	64.5 ± 6.8	72.4 ± 6.3	79.1 ± 6.9	77.6 ± 4.4	75.8 ± 4.4
NEUT%(%)	24.7 ± 2.9	25.3 ± 9.9	33.2 ± 14.0	30.3 ± 6.7	20.3 ± 5.4	14.9 ± 4.8	17.3 ± 2.6	18.6 ± 3.3
EOS%(%)	2.3 ± 0.7	2.6 ± 1.0	2.4 ± 0.7	2.3 ± 0.9	3.5 ± 1.7	2.8 ± 1.3	2.7 ± 1.6	2.7 ± 0.9
BASO%(%)	0.8 ± 0.4	2.7 ± 4.5	0.8 ± 0.4	0.9 ± 0.9	0.7 ± 0.6	0.5 ± 0.4	0.4 ± 0.1	0.6 ± 0.2
MONO%(%)	1.8 ± 0.4	1.8 ± 0.6	2.0 ± 0.7	1.7 ± 0.6	2.6 ± 1.6	2.1 ± 1.4	1.7 ± 0.6	1.9 ± 0.4
RET(%)	2.28 ± 0.43	1.91 ± 0.33	2.16 ± 0.25	1.71 ± 0.48	1.78 ± 0.43	2.23 ± 0.60	1.94 ± 0.45	2.31 ± 0.62
D182 and D183								
RBC(10^12^/L)	8.51 ± 0.39	8.80 ± 0.44	8.92 ± 0.25*	9.06 ± 0.25**	7.66 ± 0.42	7.61 ± 0.30	7.72 ± 0.30	7.43 ± 0.32
HCT(%)	46.2 ± 2.5	46.7 ± 1.9	47.3 ± 1.7	48.1 ± 1.4	42.7 ± 2.0	43.2 ± 1.6	43.7 ± 1.8	42.0 ± 1.2
MCV(fL)	54.3 ± 1.9	53.1 ± 1.0	53.1 ± 1.8	53.1 ± 1.4	55.8 ± 1.6	56.8 ± 2.2	56.7 ± 2.0	56.6 ± 1.5
HGB(g/dL)	14.8 ± 0.7	14.8 ± 0.5	15.1 ± 0.5	15.3 ± 0.4	13.9 ± 0.7	14.1 ± 0.4	14.3 ± 0.6	13.7 ± 0.3
MCH(pg)	17.4 ± 0.8	16.8 ± 0.5	16.9 ± 0.6	16.9 ± 0.5	18.1 ± 0.6	18.5 ± 0.6	18.5 ± 0.6	18.5 ± 0.5
MCHC(g/dL)	31.91 ± 0.93	31.74 ± 0.55	31.90 ± 0.41	31.92 ± 0.45	32.55 ± 0.21	32.60 ± 0.35	32.70 ± 0.36	32.70 ± 0.37
PLT(10^9^/L)	1,051 ± 198	947 ± 69	938 ± 118	943 ± 123	905 ± 135	894 ± 65	920 ± 154	917 ± 117
WBC(10^9^/L)	3.91 ± 1.60	3.14 ± 1.17	3.30 ± 1.34	3.71 ± 1.28	2.16 ± 0.88	1.90 ± 0.68	1.69 ± 0.53	1.78 ± 0.44
LYM%(%)	61.4 ± 12.4	61.2 ± 4.7	59.4 ± 9.5	57.7 ± 12.9	59.8 ± 5.8	63.3 ± 13.1	69.8 ± 5.8	68.3 ± 7.7
NEUT%(%)	30.9 ± 12.2	30.6 ± 4.5	33.0 ± 8.8	34.1 ± 12.3	31.6 ± 6.6	28.3 ± 12.4	21.8 ± 5.4*	22.0 ± 6.6*
EOS%(%)	3.9 ± 1.7	4.3 ± 1.6	4.5 ± 1.5	4.2 ± 1.4	4.4 ± 1.6	5.0 ± 2.5	5.0 ± 1.8	5.5 ± 1.6
BASO%(%)	1.8 ± 1.1	1.0 ± 0.4	0.7 ± 0.3	1.4 ± 0.6	0.9 ± 0.5	0.6 ± 0.4	0.5 ± 0.4	0.6 ± 0.8
MONO%(%)	2.1 ± 1.0	2.5 ± 1.0	1.9 ± 0.6	2.4 ± 0.7	2.7 ± 1.0	2.5 ± 1.0	2.4 ± 0.9	2.9 ± 0.9
RET(%)	2.13 ± 0.49	2.07 ± 0.43	1.82 ± 0.20	1.67 ± 0.28	2.34 ± 0.39	2.16 ± 0.73	2.16 ± 0.48	2.13 ± 0.45
End of recovery								
RBC(10^12^/L)	8.94 ± 0.67	8.76 ± 0.10	8.88 ± 0.30	8.98 ± 0.21	7.42 ± 0.13	7.50 ± 0.28	7.52 ± 0.13	7.69 ± 0.32
HCT(%)	46.1 ± 2.7	46.7 ± 1.3	47.0 ± 1.7	46.7 ± 1.7	41.0 ± 1.2	42.7 ± 1.0	41.8 ± 0.9	43.0 ± 1.8
MCV(fL)	51.6 ± 1.6	53.3 ± 0.9	52.9 ± 0.8	52.0 ± 1.5	55.2 ± 1.2	57.0 ± 1.1	55.6 ± 1.2	56.0 ± 2.5
HGB(g/dL)	14.8 ± 0.8	15.1 ± 0.5	15.2 ± 0.5	15.0 ± 0.7	13.7 ± 0.4	14.2 ± 0.4	13.8 ± 0.2	14.4 ± 0.4*
MCH(pg)	16.6 ± 0.6	17.2 ± 0.4	17.1 ± 0.3	16.7 ± 0.6	18.5 ± 0.4	18.9 ± 0.3	18.4 ± 0.5	18.7 ± 0.9
MCHC(g/dL)	32.12 ± 0.13	32.28 ± 0.24	32.30 ± 0.39	32.20 ± 0.38	33.42 ± 0.33	33.18 ± 0.40	33.10 ± 0.47	33.42 ± 0.66
PLT(10^9^/L)	1,038 ± 73	938 ± 102	1,000 ± 84	1,024 ± 70	907 ± 141	809 ± 144	835 ± 63	796 ± 72
WBC(10^9^/L)	3.09 ± 1.40	2.74 ± 0.57	3.47 ± 0.75	3.16 ± 0.53	1.54 ± 0.18	1.09 ± 0.34	1.23 ± 0.53	1.18 ± 0.31
LYM%(%)	63.8 ± 7.1	54.6 ± 13.8	54.2 ± 12.4	58.4 ± 10.8	58.1 ± 13.3	57.2 ± 7.1	54.9 ± 9.0	56.2 ± 6.3
NEUT%(%)	28.5 ± 5.7	36.8 ± 12.6	37.4 ± 12.1	31.7 ± 10.8	27.5 ± 12.4	27.1 ± 4.6	28.9 ± 8.9	27.2 ± 6.5
EOS%(%)	4.6 ± 1.8	5.6 ± 2.8	5.5 ± 1.1	6.5 ± 2.5	11.0 ± 1.3	12.8 ± 2.4	12.1 ± 3.0	13.1 ± 1.8
BASO%(%)	1.2 ± 0.7	0.9 ± 0.4	0.9 ± 0.5	1.0 ± 0.4	0.7 ± 0.7	0.4 ± 0.4	1.5 ± 1.5	0.6 ± 0.6
MONO%(%)	1.7 ± 0.6	1.9 ± 0.6	1.9 ± 0.5	2.2 ± 0.4	2.6 ± 0.7	2.2 ± 1.0	2.5 ± 1.1	2.7 ± 0.6
RET(%)	2.03 ± 0.29	1.70 ± 0.11	2.12 ± 0.10	2.03 ± 0.42	1.79 ± 0.23	2.08 ± 0.48	1.85 ± 0.09	2.23 ± 0.47

Note: The data represent Mean ± SD, *p < 0.05, **p < 0.01 *versus* the control. n represent the number of animals in each group n = 5, on Day 29; n = 5, on Day 92; n = 10, on Day 182; n = 10, on Day 183; n = 5, at the end of recovery.

In addition, significant decreases were observed in neutrophil ratio in the low-dose females (*p* < 0.05) on Day 29, and in mid-dose (*p* < 0.05) and high-dose(*p* < 0.05) females on Day 183. There was no significant difference in other hematological parameters between the DLTD-treated and the control groups. Above parameters remained within the physiological range throughout the 30-week experimental period. There is no toxicological significance in the clinical hematology in both sexes from the each dose group.

#### 3.2.3 Blood chemistry

The effect of oral administration of DLTD on blood chemistry is presented in Table 3. The serum glucose were significantly decreased in low-dose (*p* < 0.05) and mid-dose males (*p* < 0.05), and the serum triglycerides were significantly decreased in mid-dose males (*p* < 0.05) on Day 92. On Day 182 significant decrease were observed in serum urea nitrogen in low-dose males (*p* < 0.01), and in serum potassium in low-dose males (*p* < 0.05). In high-dose females, total bilirubin were significantly decreased, while serum glucose were significantly increased on day 183. There was no significant difference in other biochemistry parameters between the DLTD-treated and the control groups. Above parameters remained within the physiological range and there was neither dose response relationship nor time response relationship throughout the 30-week experimental period. There is no toxicological significance in the clinical blood chemistry in both sexes from the each dose group.

**TABLE 3:** Blood biochemistry parameters in rats treated with DLTD.

Parameter	Male				Female			
	Control	Low	Medium	High	Control	Low	Medium	High
D29								
TP(g/L)	58.2 ± 2.6	60.2 ± 1.3	61.5 ± 3.3	60.6 ± 1.5	64.2 ± 3.2	65.0 ± 1.8	63.0 ± 2.9	64.9 ± 3.5
ALB(g/L)	34.3 ± 0.9	35.4 ± 1.3	36.0 ± 1.6	35.5 ± 1.1	38.7 ± 2.0	39.2 ± 1.1	38.4 ± 1.8	39.2 ± 4.1
ALT(U/L)	30 ± 4	34 ± 3	28 ± 6	29 ± 3	23 ± 2	25 ± 2	22 ± 2	30 ± 12
AST(U/L)	99 ± 5	99 ± 9	105 ± 17	98 ± 13	90 ± 9	98 ± 16	98 ± 16	94 ± 24
ALP(U/L)	192 ± 39	186 ± 48	185 ± 33	189 ± 45	111 ± 15	102 ± 27	139 ± 18	123 ± 22
GGT(U/L)	0.466 ± 0.205	0.594 ± 0.263	0.746 ± 0.299	0.706 ± 0.437	1.014 ± 0.229	1.016 ± 0.202	1.300 ± 0.360	1.302 ± 0.390
TBIL(μmol/L)	0.6 ± 0.3	0.6 ± 0.1	0.5 ± 0.2	0.5 ± 0.3	0.6 ± 0.3	0.6 ± 0.3	0.4 ± 0.1	0.3 ± 0.1
LDH(U/L)	542.8 ± 222.0	504.8 ± 111.8	744.2 ± 377.7	514.0 ± 256.4	406.2 ± 124.0	689.3 ± 339.5	551.4 ± 285.8	484.1 ± 213.4
CK(U/L)	557 ± 196	660 ± 258	666 ± 182	626 ± 269	355 ± 100	488 ± 198	470 ± 125	442 ± 88
CKMB(U/L)	613 ± 261	526 ± 120	725 ± 320	544 ± 297	365 ± 95	642 ± 312	479 ± 249	464 ± 154
CRE(μmol/L)	21 ± 2	22 ± 3	22 ± 2	22 ± 2	28 ± 3	29 ± 4	30 ± 3	27 ± 1
UREA(μmol/L)	5.56 ± 0.75	5.61 ± 0.76	5.73 ± 0.43	5.81 ± 0.73	6.52 ± 1.02	6.55 ± 1.00	6.09 ± 0.72	5.93 ± 1.12
GLU(mmol/L)	7.64 ± 1.41	7.52 ± 1.38	8.39 ± 1.91	7.48 ± 1.50	7.63 ± 0.52	7.21 ± 0.79	7.06 ± 0.64	6.83 ± 0.56
CHO(mmol/L)	1.51 ± 0.24	1.34 ± 0.24	1.59 ± 0.26	1.49 ± 0.33	1.56 ± 0.33	1.71 ± 0.30	1.57 ± 0.17	1.56 ± 0.24
TG(mmol/L)	0.41 ± 0.18	0.28 ± 0.14	0.21 ± 0.09	0.35 ± 0.09	0.26 ± 0.09	0.23 ± 0.06	0.21 ± 0.03	0.18 ± 0.03
Na^+^(mmol/L)	146.8 ± 2.0	146.8 ± 1.1	147.0 ± 1.3	146.0 ± 0.9	145.6 ± 1.0	145.4 ± 0.6	146.0 ± 2.0	145.5 ± 1.2
K^+^(mmol/L)	4.44 ± 0.15	4.53 ± 0.17	4.87 ± 0.30	4.75 ± 0.34	4.30 ± 0.34	4.54 ± 0.27	4.49 ± 0.31	4.54 ± 0.27
Cl^−^(mmol/L)	113.5 ± 1.9	113.6 ± 1.0	113.6 ± 1.7	112.5 ± 1.7	112.6 ± 1.7	112.3 ± 1.3	114.3 ± 1.8	113.2 ± 0.3
D92								
TP(g/L)	59.1 ± 1.0	58.2 ± 1.6	57.8 ± 5.5	59.2 ± 3.3	70.1 ± 8.2	67.9 ± 5.0	69.4 ± 5.2	73.6 ± 4.1
ALB(g/L)	31.1 ± 0.7	31.3 ± 0.4	30.9 ± 2.6	31.2 ± 2.0	41.5 ± 4.6	39.6 ± 3.2	39.7 ± 4.1	41.9 ± 2.5
ALT(U/L)	40 ± 11	45 ± 19	34 ± 2	32 ± 5	107 ± 63	72 ± 61	67 ± 55	90 ± 61
AST(U/L)	122 ± 15	133 ± 43	131 ± 38	118 ± 40	228 ± 121	217 ± 142	210 ± 152	297 ± 225
ALP(U/L)	94 ± 16	85 ± 17	80 ± 22	78 ± 6	38 ± 12	33 ± 7	36 ± 4	29 ± 8
GGT(U/L)	0.592 ± 0.189	0.634 ± 0.344	0.720 ± 0.407	0.692 ± 0.736	0.732 ± 0.344	0.582 ± 0.276	0.684 ± 0.121	0.680 ± 0.153
TBIL(μmol/L)	0.6 ± 0.2	0.9 ± 0.4	0.7 ± 0.3	0.5 ± 0.4	0.8 ± 0.3	0.6 ± 0.3	0.4 ± 0.3	0.1 ± 0.6
LDH(U/L)	617.6 ± 507.8	504.8 ± 383.0	601.3 ± 370.3	825.9 ± 613.4	592.2 ± 374.6	754.5 ± 382.9	642.5 ± 452.3	645.3 ± 295.5
CK(U/L)	487 ± 346	496 ± 232	550 ± 445	674 ± 481	473 ± 150	678 ± 234	636 ± 251	602 ± 164
CKMB(U/L)	390 ± 257	366 ± 261	396 ± 316	494 ± 335	355 ± 201	440 ± 235	470 ± 384	382 ± 209
CRE(μmol/L)	26 ± 2	23 ± 2	25 ± 2	23 ± 3	30 ± 5	29 ± 2	32 ± 10	28 ± 4
UREA(μmol/L)	6.17 ± 1.02	6.27 ± 0.35	6.30 ± 0.76	7.17 ± 1.58	6.49 ± 0.89	6.66 ± 1.10	6.88 ± 1.41	5.95 ± 0.60
GLU(mmol/L)	11.71 ± 1.17	9.12 ± 0.66*	8.59 ± 0.99 *	8.38 ± 2.14	8.03 ± 1.78	7.56 ± 1.43	7.02 ± 0.72	7.99 ± 1.17
CHO(mmol/L)	1.98 ± 0.35	1.84 ± 0.28	1.69 ± 0.41	1.71 ± 0.28	2.81 ± 0.77	2.16 ± 0.71	3.01 ± 1.14	2.71 ± 0.46
TG(mmol/L)	0.43 ± 0.19	0.40 ± 0.15	0.22 ± 0.06*	0.24 ± 0.03	0.30 ± 0.06	0.23 ± 0.04	0.26 ± 0.05	0.30 ± 0.12
Na^+^(mmol/L)	154.8 ± 4.4	153.8 ± 2.7	153.2 ± 2.2	152.9 ± 4.5	148.1 ± 3.8	149.6 ± 3.1	149.5 ± 4.4	148.7 ± 2.4
K^+^(mmol/L)	5.32 ± 0.61	4.99 ± 0.47	5.16 ± 0.46	5.59 ± 0.43	4.88 ± 0.42	4.62 ± 0.30	4.57 ± 0.24	5.05 ± 0.38
Cl^−^(mmol/L)	122.5 ± 3.8	120.9 ± 2.2	120.9 ± 3.0	120.9 ± 3.5	114.5 ± 3.0	117.3 ± 3.8	115.6 ± 4.0	116.4 ± 1.7
D182 and D183								
TP(g/L)	58.9 ± 5.6	59.1 ± 2.2	59.6 ± 1.7	58.1 ± 3.8	70.5 ± 2.9	69.8 ± 5.2	71.4 ± 4.3	71.0 ± 4.4
ALB(g/L)	32.9 ± 3.1	33.6 ± 1.7	33.7 ± 0.9	32.9 ± 1.2	45.1 ± 2.7	44.0 ± 4.3	44.9 ± 3.2	44.3 ± 3.0
ALT(U/L)	36 ± 14	43 ± 29	33 ± 12	28 ± 3	77 ± 62	74 ± 73	44 ± 20	34 ± 10
AST(U/L)	162 ± 48	154 ± 51	153 ± 31	150 ± 31	221 ± 141	180 ± 117	128 ± 38	112 ± 18
ALP(U/L)	83 ± 47	73 ± 15	70 ± 12	74 ± 14	39 ± 14	41 ± 11	44 ± 18	41 ± 8
GGT(U/L)	0.684 ± 1.013	0.325 ± 0.219	0.466 ± 0.204	0.575 ± 0.358	0.565 ± 0.569	0.664 ± 0.272	0.442 ± 0.241	0.757 ± 0.596
TBIL(μmol/L)	0.7 ± 0.3	0.8 ± 0.3	0.7 ± 0.2	0.7 ± 0.1	1.1 ± 0.4	0.8 ± 0.3	0.9 ± 0.3	0.6 ± 0.2*
LDH(U/L)	1,837.3 ± 829.3	1,477.6 ± 672.6	1,678.7 ± 665.7	1,712.0 ± 653.0	1,040.1 ± 659.8	721.1 ± 338.6	567.7 ± 232.8	695.2 ± 423.2
CK(U/L)	1,124 ± 570	938 ± 499	1,074 ± 441	1,107 ± 413	679 ± 470	469 ± 134	428 ± 193	527 ± 259
CKMB(U/L)	1,731 ± 931	1,320 ± 836	1,561 ± 788	1,534 ± 683	952 ± 762	584 ± 263	440 ± 169	627 ± 410
CRE(μmol/L)	26 ± 4	23 ± 2	26 ± 4	24 ± 3	31 ± 3	32 ± 4	32 ± 4	31 ± 4
UREA(μmol/L)	6.52 ± 1.14	5.33 ± 0.27**	6.27 ± 0.83	6.01 ± 0.53	6.16 ± 1.12	7.24 ± 0.89	6.81 ± 1.05	7.02 ± 1.35
GLU(mmol/L)	13.33 ± 6.74	10.49 ± 1.21	9.44 ± 1.36	8.02 ± 1.13	7.34 ± 0.66	7.83 ± 0.94	7.72 ± 1.52	8.87 ± 1.26*
CHO(mmol/L)	2.12 ± 0.36	2.14 ± 0.28	2.00 ± 0.37	1.79 ± 0.33	3.35 ± 0.51	3.06 ± 0.91	2.78 ± 0.45	3.25 ± 0.47
TG(mmol/L)	0.53 ± 0.23	0.46 ± 0.23	0.40 ± 0.08	0.32 ± 0.11	0.44 ± 0.17	0.43 ± 0.08	0.42 ± 0.14	0.38 ± 0.08
Na^+^(mmol/L)	140.4 ± 3.2	142.0 ± 1.0	141.3 ± 1.2	141.6 ± 1.1	145.8 ± 1.9	145.4 ± 1.9	144.4 ± 1.5	144.2 ± 1.2
K^+^(mmol/L)	5.01 ± 0.40	4.65 ± 0.21*	4.99 ± 0.29	4.88 ± 0.25	4.40 ± 0.47	4.45 ± 0.29	4.14 ± 0.43	4.61 ± 0.34
Cl^−^(mmol/L)	105.9 ± 2.6	107.5 ± 1.2	107.5 ± 1.7	107.9 ± 1.2	110.1 ± 2.5	110.0 ± 2.5	108.8 ± 2.2	109.8 ± 2.3
End of recovery								
TP(g/L)	62.2 ± 1.4	61.1 ± 1.6	63.7 ± 2.3	64.9 ± 2.1	75.0 ± 4.4	73.5 ± 2.0	73.7 ± 1.9	72.3 ± 2.8
ALB(g/L)	34.5 ± 0.7	33.6 ± 1.0	34.8 ± 1.0	35.4 ± 1.3	46.5 ± 4.0	46.3 ± 1.0	46.9 ± 0.4	45.8 ± 3.3
ALT(U/L)	42 ± 9	33 ± 4	37 ± 8	35 ± 5	63 ± 36	76 ± 68	37 ± 13	44 ± 19
AST(U/L)	183 ± 13	157 ± 33	160 ± 8	173 ± 25	218 ± 185	234 ± 215	116 ± 25	115 ± 23
ALP(U/L)	74 ± 19	75 ± 8	61 ± 5	67 ± 6	31 ± 10	24 ± 12	35 ± 14	26 ± 5
GGT(U/L)	0.678 ± 0.461	0.758 ± 0.259	0.622 ± 0.423	0.804 ± 0.317	0.488 ± 0.149	0.622 ± 0.573	0.514 ± 0.369	0.564 ± 0.385
TBIL(μmol/L)	0.8 ± 0.4	0.6 ± 0.1	0.6 ± 0.1	0.7 ± 0.1	1.1 ± 0.5	1.0 ± 0.8	0.9 ± 0.4	0.8 ± 0.4
LDH(U/L)	1,882.1 ± 340.2	1,368.2 ± 664.5	1,788.4 ± 441.2	2,004.2 ± 427.7	688.0 ± 353.8	865.5 ± 466.5	593.6 ± 258.7	436.5 ± 159.1
CK(U/L)	1,280 ± 361	849 ± 348	1,245 ± 217	1,418 ± 344	444 ± 193	689 ± 244	443 ± 98	359 ± 53
CKMB(U/L)	1,767 ± 414	1,135 ± 570	1,676 ± 526	1,881 ± 581	522 ± 258	715 ± 394	444 ± 135	336 ± 109
CRE(μmol/L)	30 ± 3	30 ± 5	29 ± 7	27 ± 1	31 ± 2	29 ± 3	33 ± 6	30 ± 1
UREA(μmol/L)	6.03 ± 0.61	6.18 ± 0.43	6.04 ± 0.71	6.11 ± 0.44	6.02 ± 0.61	5.45 ± 0.39	6.25 ± 1.12	6.35 ± 0.63
GLU(mmol/L)	11.66 ± 2.58	11.97 ± 2.10	9.73 ± 1.37	9.36 ± 2.09	8.16 ± 0.98	8.07 ± 1.28	8.45 ± 0.65	7.77 ± 0.58
CHO(mmol/L)	2.21 ± 0.24	2.01 ± 0.47	2.25 ± 0.54	2.63 ± 0.66	3.35 ± 0.57	3.52 ± 0.74	3.67 ± 0.67	3.09 ± 0.28
TG(mmol/L)	0.76 ± 0.35	0.53 ± 0.18	0.62 ± 0.27	0.67 ± 0.31	0.35 ± 0.04	0.41 ± 0.10	0.38 ± 0.05	0.37 ± 0.06
Na^+^(mmol/L)	144.2 ± 2.0	144.6 ± 1.2	145.2 ± 0.6	144.4 ± 1.4	141.5 ± 1.0	142.4 ± 2.1	141.4 ± 1.7	142.9 ± 1.5
K^+^(mmol/L)	5.23 ± 0.37	5.11 ± 0.16	5.29 ± 0.38	5.35 ± 0.16	4.16 ± 0.22	4.24 ± 0.46	4.25 ± 0.44	4.28 ± 0.30
Cl^−^(mmol/L)	110.1 ± 1.5	109.7 ± 0.9	110.7 ± 0.9	110.0 ± 0.6	106.6 ± 1.2	107.5 ± 3.2	106.9 ± 2.6	108.2 ± 1.5

Note: The data represent Mean ± SD, *p < 0.05, **p < 0.01 *versus* the control. n represent the number of animals in each group n = 5, on Day 29; n = 5, on Day 92; n = 10, on Day 182; n = 10, on Day 183; n = 5, at the end of recovery.

#### 3.2.4 Organ weight and histopathological examination

Relative organ weights (organ weight/brain weight) of the 30-week treated rats are shown in Table 4. At the mid of the treatment with DLTD, the relative kidney weights in high-dose males were significantly increased in comparison to those in control males. The relative weight change of kidney was not considered of toxicological significance since this change had neither dose response and time response relationship nor consistent histopathological changes of corresponding organs, but was strongly associated with low fasting weight. At the end of the treatment and recovery period, no significance changes were observed in the relative organ weights in males and females.

**TABLE 4:** Relative weight (organ weights/body weights)(%) in rats treated with DLTD.

Parameter	Male				Female			
	Control	Low	Medium	High	Control	Low	Medium	High
D29								
Heart	0.380 ± 0.047	0.353 ± 0.029	0.373 ± 0.013	0.331 ± 0.020	0.395 ± 0.021	0.388 ± 0.033	0.384 ± 0.030	0.374 ± 0.024
Liver	2.589 ± 0.179	2.642 ± 0.126	2.707 ± 0.204	2.807 ± 0.068	2.945 ± 0.120	2.786 ± 0.193	2.873 ± 0.179	2.900 ± 0.208
Spleen	0.178 ± 0.033	0.210 ± 0.020	0.206 ± 0.014	0.189 ± 0.016	0.226 ± 0.046	0.207 ± 0.021	0.223 ± 0.014	0.208 ± 0.008
Kidney	0.754 ± 0.088	0.750 ± 0.063	0.748 ± 0.048	0.753 ± 0.026	0.731 ± 0.058	0.726 ± 0.060	0.696 ± 0.027	0.732 ± 0.047
Thymus glands	0.118 ± 0.024	0.131 ± 0.006	0.126 ± 0.016	0.136 ± 0.017	0.193 ± 0.030	0.191 ± 0.055	0.182 ± 0.014	0.188 ± 0.024
Brain	0.593 ± 0.074	0.563 ± 0.035	0.571 ± 0.024	0.563 ± 0.050	0.798 ± 0.088	0.857 ± 0.048	0.802 ± 0.049	0.866 ± 0.043
D92								
Heart	0.327 ± 0.028	0.322 ± 0.030	0.321 ± 0.035	0.337 ± 0.044	0.397 ± 0.028	0.411 ± 0.040	0.395 ± 0.060	0.393 ± 0.021
Liver	2.525 ± 0.231	2.473 ± 0.138	2.381 ± 0.263	2.502 ± 0.107	2.723 ± 0.346	2.649 ± 0.081	2.816 ± 0.201	2.899 ± 0.165
Spleen	0.129 ± 0.013	0.151 ± 0.027	0.135 ± 0.010	0.149 ± 0.022	0.188 ± 0.016	0.201 ± 0.013	0.183 ± 0.029	0.172 ± 0.018
Lungs	0.318 ± 0.021	0.325 ± 0.033	0.326 ± 0.016	0.338 ± 0.026	0.453 ± 0.039	0.483 ± 0.020	0.473 ± 0.033	0.457 ± 0.014
Kidney	0.605 ± 0.025	0.639 ± 0.045	0.664 ± 0.058	0.762 ± 0.093^**^	0.671 ± 0.054	0.644 ± 0.076	0.642 ± 0.077	0.686 ± 0.092
Adrenal glands	0.013 ± 0.001	0.013 ± 0.003	0.013 ± 0.003	0.014 ± 0.002	0.030 ± 0.009	0.030 ± 0.008	0.029 ± 0.003	0.030 ± 0.006
Thymus glands	0.063 ± 0.015	0.079 ± 0.017	0.064 ± 0.008	0.070 ± 0.013	0.099 ± 0.019	0.099 ± 0.006	0.114 ± 0.017	0.098 ± 0.008
Brain	0.388 ± 0.009	0.415 ± 0.030	0.408 ± 0.035	0.433 ± 0.035	0.695 ± 0.019	0.782 ± 0.064	0.730 ± 0.061	0.725 ± 0.038
Testis/Ovary	0.660 ± 0.054	0.679 ± 0.027	0.667 ± 0.058	0.729 ± 0.047	0.051 ± 0.011	0.053 ± 0.016	0.048 ± 0.013	0.047 ± 0.009
Epididymismis/uterus	0.268 ± 0.014	0.303 ± 0.036	0.277 ± 0.031	0.317 ± 0.034	0.245 ± 0.061	0.233 ± 0.052	0.242 ± 0.049	0.285 ± 0.086
D182 and D183								
Heart	0.302 ± 0.035	0.295 ± 0.029	0.290 ± 0.031	0.292 ± 0.028	0.375 ± 0.030	0.353 ± 0.036	0.379 ± 0.059	0.372 ± 0.029
Liver	2.659 ± 0.423	2.480 ± 0.165	2.473 ± 0.179	2.397 ± 0.224	2.627 ± 0.173	2.672 ± 0.256	2.767 ± 0.234	2.843 ± 0.406
Spleen	0.146 ± 0.022	0.134 ± 0.012	0.135 ± 0.013	0.140 ± 0.021	0.175 ± 0.029	0.163 ± 0.020	0.170 ± 0.032	0.158 ± 0.017
Lungs	0.315 ± 0.046	0.310 ± 0.029	0.323 ± 0.025	0.323 ± 0.027	0.463 ± 0.042	0.439 ± 0.040	0.438 ± 0.045	0.434 ± 0.044
Kidney	0.636 ± 0.181	0.624 ± 0.051	0.616 ± 0.067	0.686 ± 0.054	0.630 ± 0.056	0.604 ± 0.055	0.628 ± 0.031	0.631 ± 0.057
Adrenal glands	0.011 ± 0.002	0.011 ± 0.002	0.011 ± 0.001	0.011 ± 0.001	0.028 ± 0.006	0.024 ± 0.005	0.027 ± 0.004	0.025 ± 0.003
Thymus glands	0.056 ± 0.013	0.054 ± 0.008	0.059 ± 0.015	0.054 ± 0.012	0.077 ± 0.021	0.073 ± 0.012	0.074 ± 0.013	0.074 ± 0.015
Brain	0.357 ± 0.052	0.354 ± 0.027	0.374 ± 0.042	0.390 ± 0.045	0.660 ± 0.073	0.681 ± 0.089	0.679 ± 0.071	0.675 ± 0.066
Testis/Ovary	0.591 ± 0.105	0.618 ± 0.050	0.623 ± 0.085	0.643 ± 0.058	0.046 ± 0.009	0.045 ± 0.014	0.056 ± 0.014	0.043 ± 0.011
Epididymismis/uterus	0.265 ± 0.038	0.260 ± 0.036	0.275 ± 0.037	0.274 ± 0.034	0.234 ± 0.040	0.236 ± 0.052	0.268 ± 0.081	0.230 ± 0.046
End of recovery								
Heart	0.273 ± 0.041	0.288 ± 0.041	0.285 ± 0.025	0.299 ± 0.029	0.372 ± 0.057	0.359 ± 0.049	0.374 ± 0.012	0.392 ± 0.052
Liver	2.331 ± 0.094	2.490 ± 0.509	2.440 ± 0.095	2.485 ± 0.178	2.802 ± 0.269	2.638 ± 0.264	2.681 ± 0.207	2.703 ± 0.183
Spleen	0.126 ± 0.016	0.123 ± 0.021	0.146 ± 0.028	0.127 ± 0.025	0.148 ± 0.020	0.155 ± 0.012	0.154 ± 0.020	0.166 ± 0.017
Lungs	0.300 ± 0.017	0.304 ± 0.021	0.307 ± 0.012	0.306 ± 0.037	0.416 ± 0.045	0.404 ± 0.043	0.419 ± 0.018	0.440 ± 0.018
Kidney	0.545 ± 0.047	0.607 ± 0.058	0.570 ± 0.068	0.605 ± 0.039	0.641 ± 0.041	0.642 ± 0.123	0.614 ± 0.045	0.651 ± 0.036
Adrenal glands	0.010 ± 0.001	0.010 ± 0.002	0.012 ± 0.001	0.010 ± 0.002	0.026 ± 0.004	0.024 ± 0.003	0.022 ± 0.001	0.027 ± 0.003
Thymus glands	0.047 ± 0.022	0.060 ± 0.013	0.047 ± 0.018	0.051 ± 0.014	0.078 ± 0.017	0.089 ± 0.017	0.082 ± 0.007	0.074 ± 0.013
Brain	0.331 ± 0.045	0.346 ± 0.015	0.347 ± 0.023	0.377 ± 0.057	0.639 ± 0.054	0.640 ± 0.096	0.657 ± 0.010	0.678 ± 0.046
Testis/Ovary	0.576 ± 0.064	0.600 ± 0.058	0.641 ± 0.072	0.567 ± 0.131	0.039 ± 0.005	0.038 ± 0.007	0.043 ± 0.011	0.047 ± 0.011
Epididymismis/uterus	0.258 ± 0.034	0.260 ± 0.028	0.262 ± 0.018	0.236 ± 0.027	0.255 ± 0.047	0.245 ± 0.025	0.240 ± 0.029	0.233 ± 0.019

Note: The data represent Mean ± SD, **p < 0.01 *versus* the control. n represent the number of animals in each group n = 5, on Day 29; n = 5, on Day 92; n = 10, on Day 182; n = 10, on Day 183; n = 5, at the end of recovery.

**FIGURE 3:**
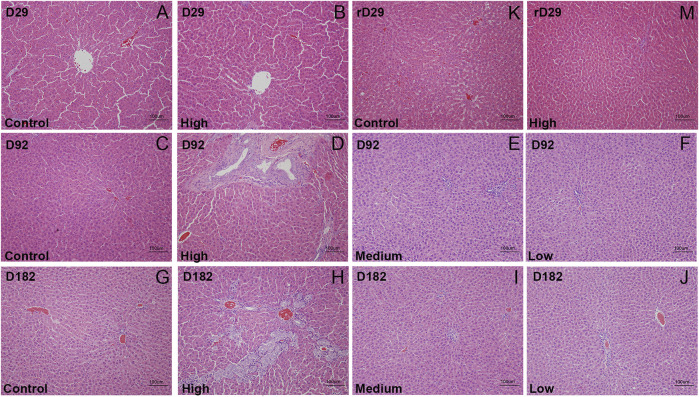
The histopathological analysis (H&E staining × 200) of liver of the control group and high-dose group were performed at D29 **(A and B)**, D92 **(C and D)**, D182**(G and H)** and rD29**(K and M)**, of the midium-dose group and low-dose group were performed at D92 **(E and F)** and D182 **(I and J)**, respectively.

After repeated oral administration of DLTD for 13 and 26 weeks, mild hypertrophy and hyperplasia of hepatic interlobular bile ducts were detected (3/10 on 13 weeks, 9/20 on 26 weeks, respectively) in some rats at high dose, but no significant changes were observed in liver at low dose and medium dose. At the end of the recovery period, the above changes in liver were eliminated and the tissue structure returned to normal Figure 3. DLTD had no obvious toxic damage to other organs and tissues.

### 3.3 Differential gene expression, GO and KEGG analysis

**FIGURE 4:**
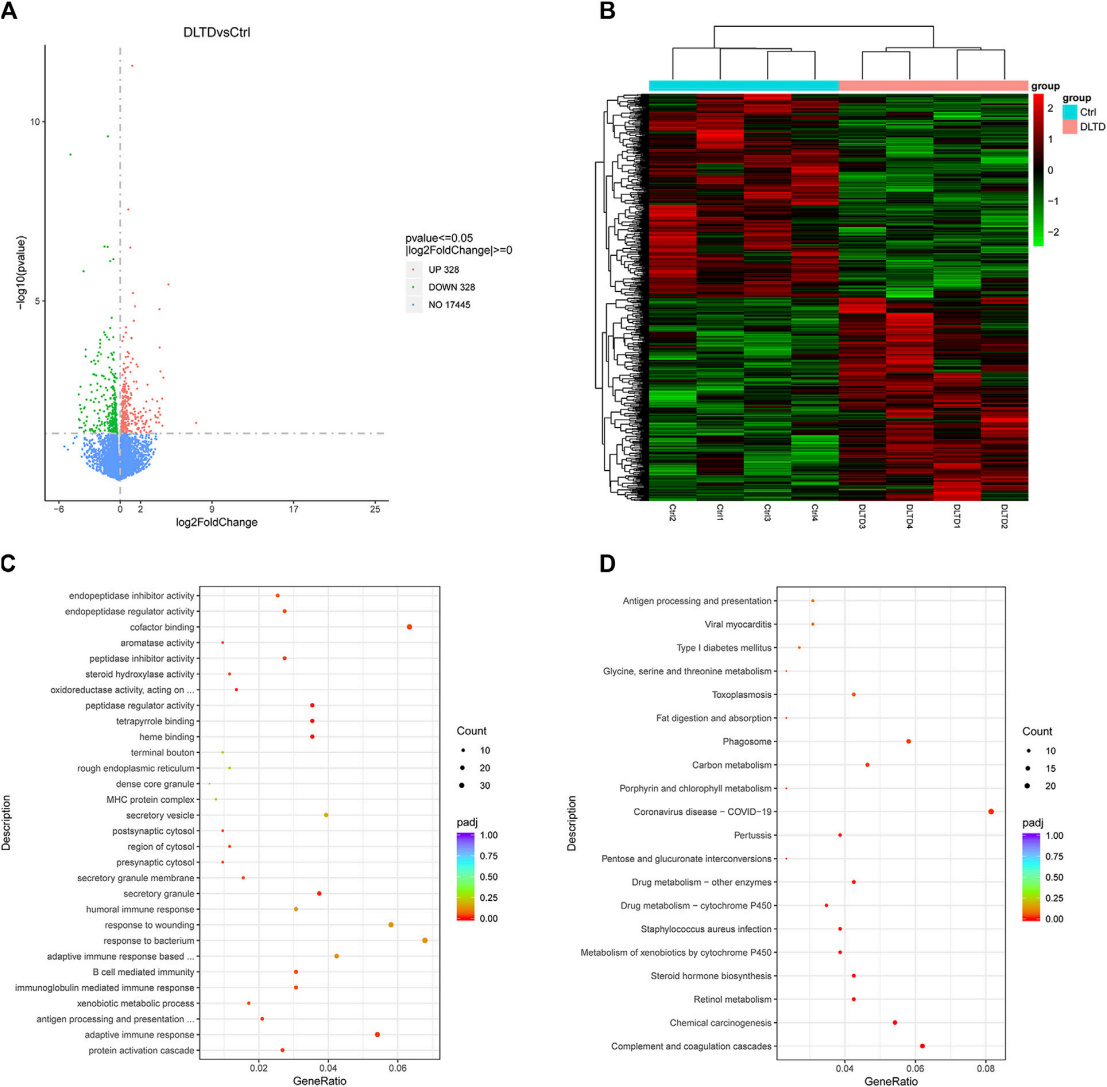
Differential gene expression, GO and KEGG Analysis of liver between the control group and DLTD group. **(A)** The number of genes upregulated and downregulated by DLTD. **(B)** Heatmap of differentially expressed genes between the control group and DLTD group. **(C)** Gene ontology (GO) enrichment and **(D)** Kyoto Encyclopedia of Genes and Genomes (KEGG) pathway enrichment analysis of differentially expressed genes between the control group and DLTD group.

To go further and explore the possible roles of DLTD on the rat liver, an RNA-seq analysis was conducted. We analyzed the gene expression profiles of the control group and high-dose group rats at the end of the administration period. Six hundred and fifty-six differentially expressed genes (DEG) were found according to the criteria of |log2 fold changes| ≥ 0 and *p*-value ≤ 0.05. Among the 656 DEGs, DLTD upregulated 328 genes and downregulated 328 genes in the liver tissue (Figure 4A). Figure 4B showed the heatmaps of the distributions of the DEGs. To characterize the potential functional pathways altered by DLTD, GO and KEGG pathway enrichment analysis were performed. The GO analysis identified 21 GO terms that were significantly enriched (*p*-value < 0.05, Figure 4C), including terms related to the biological process, cellular components and molecular function. The biological process analysis showed that the DEGs were related to protein activation cascade, adaptive immune response, antigen processing and presentation of peptide, xenobiotic metabolic process, immunoglobulin mediated immune response, B cell mediated immunity. The cellular components analysis showed that the DEGs were related to secretory granule, secretory granule membrane, presynaptic cytosol, region of cytosol, postsynaptic cytosol. The molecular function analysis showed that the DEGs were related to heme binding, tetrapyrrole binding, peptidase regulator activity, oxidoreductase activity, steroid hydroxylase activity, peptidase inhibitor activity, aromatase activity, cofactor binding, endopeptidase regulator activity, endopeptidase inhibitor activity. There were 15 pathways with a *p*-value < 0.05 figured out by the KEGG pathway analysis of the DEGs shown in Figure 4D. The significantly enriched pathways were related to complement and coagulation cascades, chemical carcinogenesis, retinol metabolism, steroid hormone biosynthesis, metabolism of xenobiotics by cytochrome P450, *staphylococcus aureus* infection, drug metabolism-cytochrome P450, enzymes drug metabolism-other enzymes, pentose and glucuronate interconversions, pertussis, coronavirus disease-COVID-19, porphyrin and chlorophyll metabolism, carbon metabolism, phagosome, fat digestion and absorption. In conclusion, we focused our research on the effect of DLTD on hepatic drug enzymes.

### 3.4 Validation of RNA-sequencing results by quantitative real-time PCR and western blot

**FIGURE 5:**
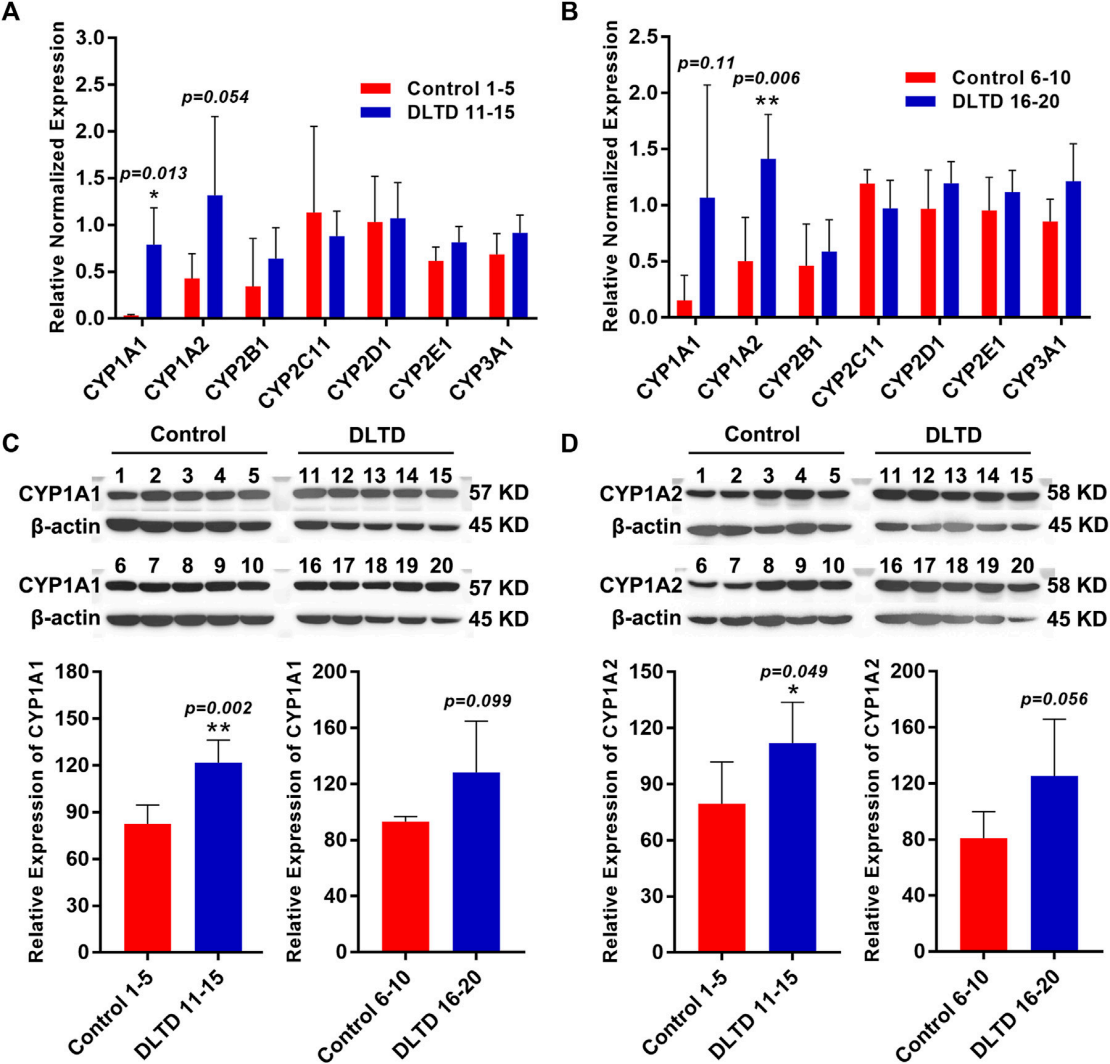
Validation the differential expression of CYP450 enzyme in the control group and DLTD group. **(A)** The mRNA expressions of CYP1A1, CYP1A2, CYP2B1, CYP2C11, CYP2D1, CYP2E1, CYP3A1 was detected in Control 1–5 and DLTD 11–15 by quantitative real-time PCR. **(B)** The mRNA expressions of CYP1A1, CYP1A2, CYP2B1, CYP2C11, CYP2D1, CYP2E1, CYP3A1 was detected in Control 6–10 and DLTD 16–20 by quantitative real-time PCR. **(C)** The protein level of CYP1A1 was detected in Control 1–5 and DLTD 11–15 or in Control 6–10 and DLTD 16–20 by western blot. **(D)** The protein level of CYP1A2 was detected in Control 1–5 and DLTD 11–15 or in Control 6–10 and DLTD 16–20 by western blot. The statistical significance of differences was calculated by *t*-test. *p < 0.05, **p < 0.01 *versus* the control.

RNA-sequencing results showed that cytochrome P450 enzymes (CYPs) including CYP1A1, CYP2B1, CYP1A2, CYP4A3, CYP3A9 and CYP3A18 were up-regulated in the high-dose group, and Log2 fold change values are 3.97, 1.57, 0.92, 0.51, 0.50 and 0.48 respectively. To confirm the results of the RNA-seq, we performed quantitative real-time PCR and western blot assays. Consistent with RNA-seq data, the mRNA expressions of CYP1A1 (Control 1–5 vs. DLTD 11–15, *p* = 0.013; Control 6–10 vs. DLTD 16–20, *p* = 0.11, Figure 5 A and B) and CYP1A2 (Control 1–5 vs. DLTD 11–15, *p* = 0.054; Control 6–10 vs. DLTD 16–20, *p* = 0.006, Figure 5A,B); the protein level of CYP1A1 (Control 1–5 vs. DLTD 11–15, *p* = 0.002; Control 6–10 vs. DLTD 16–20, *p* = 0.99, Figure 5C) and CYP1A2 (Control 1–5 vs. DLTD 11–15, *p* = 0.049; Control 6–10 vs. DLTD 16–20, *p* = 0.056, Figure 5D) were upregulated significantly in liver tissue of high dose group compared to control group (Figure 5).

## 4 Discussion

There are few information and studies regarding the toxicity of DLTD. The present study aimed to assess the toxicity and explore the mechanism of toxicity of the DLTD prior to clinical trials. The acute toxicity study demonstrated that there were no mortality or meaningful adverse changes in mice following DLTD administration. Meaningful toxicity changes in chronic toxicity studies were mild hypertrophy and hyperplasia of hepatic interlobular bile ducts were detected in some rats at high dose, and the above changes in liver were eliminated and the tissue structure returned to normal at the end of the recovery period. Based on the chronic toxicity study and three reports of drug-induced liver injury caused by DLTD, we further focused on the impact of DLTD on the liver. Four male rats were selected from the control group and the high-dose group respectively, and their liver tissues were analyzed by transcriptome. The results of transcriptome sequencing showed that DLTD had a significant effect on CYPs in rat liver, especially CYP1 subtype.

In human body, CYPs as a terminal oxygenase, are responsible for the oxidation-reduction reaction process of endogenous substrates, exogenous compounds and 90% of common drugs, and plays an important role in ensuring drug efficacy and controlling drug toxicity. So far, fifty-seven human genes coding for various CYPs have been identified. Based on the similarity of amino acid sequences, CYPs genes can be mainly divided into CYP1, CYP2 and CYP3 subfamilies(Stipp and Acco, 2021). CYP1 family comprises three members, namely CYP1A1, CYP1A2, and CYP1B1. CYP1A1 can catalyze the biotransformation of polycyclic aromatic hydrocarbons, aromatic amines, and polychlorinated biphenyls into polar compounds, so that can be conjugated to soluble compounds suitable for excretion by urine or bile. As an important metabolic enzyme in the body, CYP1A1 participates in a variety of physiological processes, such as immune response, oxidative stress, mutation induction and so on; and related to a variety of diseases, including various tumors, acute and chronic inflammation, lung injury, atherosclerosis, pathogen infection, etc(Gideon et al., 2012; Hussain et al., 2014; Gdula-Argasińska et al., 2016; Schiering et al., 2017). CYP1A2 is one of the major CYPs in human liver (∼13%–15%) and metabolizes of more than 20 drugs, such as caffeine, theophylline, warfarin, clozapine, phenacetin, tacrine, propranolol, and mexiletine(Zhou et al., 2009). CYP1A2 also plays an essential role in the metabolism of endogenous substrates and environmental toxins, as well as the activation of some precarcinogens. CYP1A2 enzyme activity varies significantly from individual to individual and can be affected by smoking, drugs, genes, and other factors(Guo et al., 2021).

Liu et al. found that doxycycline and azithromycin and their combination could cause drug-induced oxidative liver injury by increasing the expression levels of CYP1A1 and CYP1B1 (Liu et al., 2019). Hussain’s reserch confirmed that induction of CYP1A1, CYP1A2 and CYP1B1 increased oxidative stress and inflammation in the lung and liver tissues of rats exposed to incense smoke (Hussain et al., 2014). The metabolic enzyme system of CYPs were also the essential place for the metabolism of most components of traditional Chinese medicine to exert pharmacological and toxicological effects. When the activity of CYPs is inhibited or induced, drug interactions and adverse reactions are probably occur, which will lead to hepatotoxicity (Liao and Chen, 2012). Xanthii Fructus induced the activity of CYP1A2 and CYP3A4 enzymes, after processing, it could reduce the induction of CYP1A2 and CYP3A4 enzymes, and at the same time reduce liver damage (Huang et al., 2022). Emodin could induce intracellular oxidative stress and endoplasmic reticulum stress through AHR-CYP1A1 pathway, and then mediated apoptosis pathway leading to hepatocyte injury (Wang, 2018). The Polygoni Multiflori Radix and gallic acid caused significant damage to the liver function of mice by inducing the expression of CYP1A1, CYP3A4 mRNA and protein, and thereby activating the expression of the apoptosis factor Caspase-3 (Wang, 2020). Rhizoma dioscoreae bulbiferae could cause liver injury by upregulating the expression of CYP1A2 and CYP2E1 proteins in rat liver tissue (Liu, 2014).

There are many reports on drug-induced hepatobiliary hyperplasia. Drugs can cause biliary tract irritation or injury through bile excretion or the action of its metabolites (Hickling et al., 2010). Bile duct hyperplasia usually begins with bile duct epithelial injury and inflammation, and finally appears bile duct hyperplasia. At this time, inflammation, fibroblasts and bile duct hyperplasia appear in the portal duct area, and sometimes the proliferated bile duct extends to the liver lobular parenchyma, and may also be accompanied by oval cell proliferation (Gopinath et al., 1987). It has been confirmed that DLTD could cause mild hypertrophy and hyperplasia of interlobular bile ducts, and that DLTD could significantly up-regulate of CYP1A1 and CYP1A2 expression. Therefore, we speculate that the causes of mild liver injury caused by DLTD may include two aspects: on the one hand, the increased expression of CYP1A1 and CYP1A2 will accelerate the metabolism of drugs in the liver, produce more active intermediate substances to stimulate the bile duct system, and cause bile duct hyperplasia; on the other hand, the up-regulation of CYP1A1 and CYP1A2 expression can aggravate the oxidative stress and inflammatory stimulation of the hepatobiliary system, and then cause the damage of the hepatobiliary system. In addition, given the genetic polymorphisms of CYP1A1 and CYP1A2 (Vibhuti et al., 2010), high attention to the specific constitution, close observation to understand the condition, to the elderly, infants, gestating period, and liver function decline in patients with drugs, should be monitored regularly, ensure that the incidence of serious adverse reactions was reduced and drug safety.

## Data Availability

The datasets presented in this study can be found in online repositories. The names of the repository/repositories and accession number(s) can be found below: GEO, the series record number is GSE212036.
